# PPAR Regulation of Inflammatory Signaling in CNS Diseases

**DOI:** 10.1155/2008/658520

**Published:** 2008-07-28

**Authors:** John J. Bright, Saravanan Kanakasabai, Wanida Chearwae, Sharmistha Chakraborty

**Affiliations:** ^1^Neuroscience Research Laboratory, Methodist Research Institute, Clarian Health, Indianapolis, IN 46202, USA; ^2^Department of Medicine, Indiana University School of Medicine, Indianapolis, IN 46202, USA

## Abstract

Central nervous system (CNS) is an immune privileged site, nevertheless inflammation associates with many CNS diseases. Peroxisome proliferator-activated receptors (PPARs) are a family of nuclear hormone receptors that regulate immune and inflammatory responses. Specific ligands for PPAR*α*, *γ*, and *δ* isoforms have proven effective in the animal models of multiple sclerosis (MS), Alzheimer's disease, Parkinson's disease, and trauma/stroke, suggesting their use in the treatment of neuroinflammatory diseases. The activation of NF-*κ*B and Jak-Stat signaling pathways and secretion of inflammatory cytokines are critical in the pathogenesis of CNS diseases. Interestingly, PPAR agonists mitigate CNS disease by modulating inflammatory signaling network in immune cells. In this manuscript, we review the current knowledge on how PPARs regulate neuroinflammatory signaling networks in CNS diseases.

## 1. INTRODUCTION

The central nervous system (CNS) was thought to be an immune privileged site due to the ability of blood-brain-barrier (BBB) to shield immune cell entry and protect from the constantly changing circulatory milieu. Nevertheless, activated immune cells readily traverse the BBB, secrete inflammatory cytokines, and mediate many CNS diseases. Neuroinflammatory diseases present major challenges to the health care system and impose substantial economic costs around the world. Current treatments targeting clinical symptoms of CNS diseases have modest therapeutic values in patients. Significant progress has been made in recent years in developing therapeutic strategies for the treatment of neuroinflammatory diseases.

## 2. NEUROINFLAMMATORY DISEASES

The innate and adaptive immunity evoked during infection in the CNS often leads to the development of neuroinflammatory diseases [1–3]. The mounting evidence suggests that neuroinflammatory diseases such as multiple sclerosis (MS), Alzheimer's disease (AD), trauma, and ischemia/stroke can occur in the absence of infection. MS is an inflammatory demyelinating disease of the CNS with clinical symptoms ranging from pain to paralysis and the patients becoming wheel-chair bound for rest of their lives [4]. Although the etiology of MS is not known, it is generally viewed as a neural antigen-specific T cell-mediated autoimmune disease [4–6]. Experimental allergic encephalomyelitis (EAE) is an autoimmune disease model of MS, commonly used to study the mechanism of disease pathogenesis and to test the efficacy of potential therapeutic agents for the treatment of MS. In AD, the deposition of beta-amyloid (A*β*) and plaque formation in the CNS associate with inflammation resulting in neuronal death, progressive deterioration of cognitive functions, and memory loss [7, 8]. Traumatic brain injury (TBI), spinal cord injury, and ischemic stroke also display neuroinflammation associated secondary tissue damage in the CNS [9, 10]. The pathogenesis of neuroinflammatory diseases involves the orchestrated interaction of immune cells resulting in tissue injury to the CNS [6, 11]. Although the exact mechanisms are not known, recent evidence suggests the use of peroxisome proliferator-activated receptor (PPAR) agonists in the treatment of neuroinflammatory diseases.

## 3. PPAR ISOFORMS AND THEIR LIGANDS

**Figure 1:**
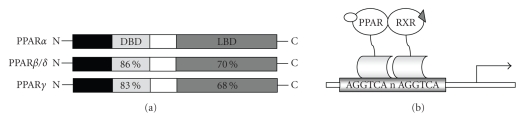
(a) Functional domains of PPAR isoforms. N, N-terminus; DBD: DNA-binding domain; LBD: ligand-binding domain. The numbers represent percentage identity to human PPAR*α*. (b) PPAR/RXR binds to PPREDR-1 promoter regions. Binding of agonists leads to heterodimerization, recruitment of coactivator and transcriptional activation of target genes.

PPAR is a family of ligand-dependent nuclear hormone receptor transcription factors that play key roles in the regulation of immune and inflammatory responses [12]. Structure-function analyses revealed that PPARs are composed of a DNA-binding domain (DBD) linked to the C-terminal ligand-binding domain (LBD) by a hinge region (Figure 1) [13, 14]. PPARs stimulate gene expression through binding to peroxisome-proliferator response elements (PPREs), present in the promoter regions of the target genes. In the absence of ligands, the heterodimers physically associate with corepressors and suppress gene transcription [14, 15]. Upon ligand binding, the coactivators replace corepressors and activate gene expression [16, 17]. PPAR*α*, PPAR*β*/*δ*, and PPAR*γ* are three structurally homologous isotypes found in various species which display distinct physiological and pharmacological functions [18]. The PPAR*α* is expressed in liver, kidney, intestine, heart, skeletal muscle, adrenal gland, pancreas, and brain. PPAR*α* is involved in acetylcholine metabolism, excitatory neurotransmission, and oxidative stress defense [19]. PPAR*α* also regulates lipid metabolism and energy homeostasis through its ability to stimulate the breakdown of fatty acids and cholesterol, driving gluconeogenesis and reduction in serum triglyceride levels [19]. While polyunsaturated fatty acids activate all three isoforms of PPARs with different affinities, each isotype has its own ligand binding property [20]. Fibrates, WY14643, and GW7647 are PPAR*α* agonists commonly used for the treatment of hypertriglyceridemia [19].

PPAR*β*/*δ* is ubiquitously expressed in all cell types including immature oligodendrocytes and promotes differentiation and myelination in the CNS [21–23]. PPAR*β*/*δ* null mice show an altered myelination of corpus callosum, suggesting its role in brain function [24]. PPAR*β*/*δ* regulates transcriptional activation of Acyl-CoA synthetase 2, a key enzyme in fatty acid utilization, suggesting its role in lipid metabolism in the brain. Prostagladin I_2_, GW0742, GW501516, and GW7842 are PPAR*β*/*δ* agonists which induce fatty acid oxidation in muscle [25]. PPAR*γ* expression is detected in adipose tissue intestinal mucosa, retina, skeletal muscle, heart, liver, and lymphoid organs [26]. PPAR*γ* is expressed in microglia and astrocytes and regulates inflammation in the CNS [27, 28]. Eicosanoids and prostaglandin J2 (15d-PGJ2) are the naturally occurring PPAR*γ* ligands, and thiazolidinedones (TZDs) including pioglitazone (Actos) and rosiglitazone (Avandia) are Food and Drug Administration (FDA) approved synthetic drugs for the treatment of type II diabetes [29]. Recent studies have shown the use of PPAR agonists in the treatment of many neuroinflammatory diseases.

## 4. THERAPEUTIC EFFECTS OF PPAR AGONISTS IN CNS DISEASES

The therapeutic effects of PPAR agonists have been tested in many different neuroinflammatory diseases (Table 1). The use of PPAR*γ* agonists in the treatment of MS has been tested in EAE model by different groups [30–33]. In vivo treatment with synthetic PPAR*γ* ligand, troglitazone, ameliorates EAE by reducing the infiltration of leukocytes in the CNS [34]. Two other studies also showed that in vivo treatment with PPAR*γ* ligands, 15d-PGJ_2_ and ciglitazone, ameliorates EAE [30, 31]. Oral treatment with pioglitazone inhibits chronic progressive and relapsing forms of EAE even when administered at the peak of disease [35, 36], suggesting their use of PPAR*γ* agonists in the treatment of MS. PPAR*γ*-deficient heterozygous mice develop an exacerbated EAE with increased CNS inflammation and demyelination [37]. A recent report also showed that PPAR*γ* antagonists, bisphenol A diglycidyl ether (BADGE), and 2-chloro-5 nitro-N-(4 pyridyl) benzamide (T007) reversed the inhibition of EAE by PPAR*γ* agonists, further suggesting the physiological role of PPAR*γ* in the pathogenesis of EAE [38].

Epidemiological studies suggest a reduced risk of AD among the users of nonsteroidal anti-inflammatory drugs (NSAID) [39, 40]. Treatment with pioglitazone and rosiglitazone significantly reduced the lesion size, motor neuron loss, myelin loss, astrogliosis, microglial activation, and chronic thermal hyperalgesia in spinal cord injury [41]. In a rat model of AD induced by cortical A*β* injection, ciglitazone and pioglitazone suppressed the clinical symptoms significantly. In the amyloid precursor protein (APP) transgenic model of AD, treatment with pioglitazone reduced the plaque burden by affecting the production, clearance, and homeostasis of A*β* in the CNS [42]. A clinical trial involving 500 AD patients showed significant improvement in cognition following treatment with rosiglitazone for 6 months, suggesting its use in the treatment of AD [43]. Recent evidence also suggests that NSAIDs such as ibuprofen may delay or prevent the development of Parkinson's disease (PD) [44, 45]. Moreover, PPAR*γ* is expressed in the CNS of 1-methyl-4-phenyl-1,2,3,6-tetrahydropyridine (MPTP)-induced model of PD [24] and treatment with pioglitazone protected the animals from neuronal cell death [46]. Similar results were also generated using lipopolysaccharide (LPS)-induced inflammation model of dopaminergic neurodegeneration in rat, where pioglitazone treatment effectively reduced inflammation, oxidative stress, and restored mitochondrial function [47]. Treatment with pioglitazone also extends the survival of superoxide dismutase-1 (SOD1-G93A) transgenic animal model of amylotrophic lateral sclerosis (ALS) [36, 48–51].

The effects of PPAR agonists in reducing deleterious inflammatory responses suggest their use in the treatment of trauma, spinal cord injury, and stroke. Experimental evidence suggests that the Pro12Ala polymorphism of PPAR*γ*2 is associated with a reduced risk for ischemic stroke [52] and treatment with TZDs and 15d-PGJ2 cause neuroprotection in animal models of stroke. Treatment with PPAR*γ* agonists also reduce the infarct volumes and improve sensorimotor function in a rodent model of middle cerebral artery occlusion (MCAO) [53, 54]. Similar effects were observed following oral or intracerebrovascular administration of PPAR*γ* agonists [55, 56]. TZD-unrelated PPAR*γ* agonist L-796,449 decreases infarct size and improves neurological scores after MCAO in the rat brain [57]. Treatment with PPAR*γ* antagonist T0070907 increased the infarct size and reversed rosiglitazone-induced protection after stroke. A small clinical trial has revealed improved functional recovery after stroke in diabetic patients receiving pioglitazone or rosiglitazone compared to patients not receiving TZD therapy [58]. A recent clinical trial demonstrated that pioglitazone significantly reduced the combined risk of myocardial infarction and stroke-associated death in high-risk patients with type 2 diabetes [59].

Recent studies have demonstrated the beneficial effects of PPAR*α* agonists in the treatment of neuroinflammatory diseases. Oral treatment with gemfibrozil protects mice from EAE [60]. The tyrosine hydroxylase (TH)-positive SNpc cells express PPAR*α* and in vivo treatment with PPAR*α* agonist, fenofibrate, protects mice from MPTP-induced inflammation and neuronal loss. In vivo treatment with PPAR*α* agonist, fenofibrate and WY-14643, reduced the infarct size in mouse models of stroke [61, 62]. This effect was absent in PPAR*α* deficient mice, reinforcing receptor dependency of the observed effects . Treatment with PPAR*α* agonist, fenofibrate, decreases the neurological deficit induced by traumatic brain injury (TBI) caused by lateral fluid percussion of brain in rats [63]. Fenofibrate also reduces brain edema and ICAM-1 expression and induces neurological recovery associated with a reduction of the brain lesion. Anti-inflammatory therapies showed neuroprotective effects after spinal cord injury in rodents [64, 65]. Moreover, oral treatment with selective PPAR*β*/*δ* agonist GW0742 exerted beneficial effects in the MOGp35-55-induced EAE model [66]. GW0742 reduced the severity of EAE even when administered at the peak of clinical disease [66]. PPAR *β*/*δ* null mice exhibit significantly greater infarct sizes than wild type animals suggesting its role in stroke [67].

## 5. PPAR AGONISTS REGULATE INFLAMMATORY CYTOKINES IN CNS DISEASES

The anti-inflammatory effects of PPAR*γ* agonists have been extensively studied in CNS diseases (Table 2). While the inflammatory cytokines, IL-1*β*, IL-6, TNF*α*, IL-12, IL-23, IL-27, IFN*γ*, and IL-17, mediate the pathogenesis of CNS diseases, anti-inflammatory cytokines, IL-4, IFN*β*, TGF*β*, and IL-10, confer recovery in MS and its animal model, EAE [68–70]. In EAE model of MS, PPAR*γ* agonists decrease the TNF*α* mRNA expression in antigen-specific T cell in vitro [71]. Other studies have shown that 15d-PGJ_2_ inhibits EAE in association with inhibition of T-cell proliferation and secretion of inflammatory cytokines including IFN*γ*, IL-10, and IL-4 in culture [31–34]. PPAR*γ* agonists, 15d-PGJ2 and ciglitazone, block IL-12 signaling through Jak-Stat pathway leading to Th1 differentiation in T cells. Pioglitazone also suppresses IFN*γ* secretion in spleen T cells following stimulation with MOGp35-55 in vitro [30]. The activation of resident glial cells and infiltration of leukocytes contribute to demyelination in EAE and MS. The chemokines and chemokine receptors promote the trafficking and entry of immune cells across blood-brain barrier into the CNS in EAE and MS [71–74]. Whereas, PPAR*γ* agonists, troglitazone and pioglitazone, reduce the expression of MCP1 [33], IP-10 (CXCL3), MIG, I-TAC, MIP1*α*, and RANTES [27] that contribute to reduced infiltration of immune cells in the MOG-induced EAE [73, 74]. Moreover, the surface molecules such as MHC class II, CD40, CD28, and ICAM enhance the disease pathogenesis and CTLA4 inhibits EAE/MS [75]. Negative regulation of adhesion molecules may also account for reduced brain infiltration observed in PPAR*γ* agonists treated EAE mice.

The immunomodulatory effects of PPAR*γ* agonists were tested in human peripheral blood mononuclear cells (PBMCs). In vitro treatment of cells with pioglitazone, ciglitazone, and GW347845 abolished proliferation and cytokine secretion accompanied by DNA condensation and down-regulation of bcl-2, suggesting the induction of apoptosis in activated T lymphocytes. MS patients showed a decrease in the expression of PPAR*γ* in immune cells and a reduction in the anti-inflammatory effects of pioglitazone when compared to healthy controls [35, 36, 76]. However, treatment of immune cells derived from diabetic patients with pioglitazone in vitro or by oral treatment in vivo increased the expression and DNA-binding activity of PPAR*γ* [77]. In MS patients, pioglitazone increased the DNA binding activity of PPAR*γ* and decreased the NF-*κ*B activity by increasing I*κ*B*α*. Activated microglial cells were significantly reduced at sites of neurodegeneration in pioglitazone-treated SOD1-G93A mice, as were the protein levels of COX-2 and iNOS. The mRNA levels of the suppressor of cytokine signaling 1 and 3 genes were also increased by pioglitazone [48], but their functional significance is not well known.

In vivo treatment with PPAR*γ* agonists suppresses A*β*-evoked microglial activation and inflammatory cytokine expression, iNOS expression and NO production, and inhibition of COX-2 in A*β*-evoked animal models of AD and APP [42]. PPAR*γ* agonists also suppress A*β*-mediated activation of microglia in vitro [40–43]. The expression of iNOS in neurons resulted in neuronal cell death which was prevented by activation of PPAR*γ* in vitro and in vivo [42, 43]. Neuroinflammatory changes accompanied by activation of microglia and astrocytes and expression of TNF*α*, IL -1*β*, and iNOS play a pivotal role in PD [46]. An increase in infiltrating CD8+ T lymphocytes and IFN*γ*+ cells were also reported in the CNS with PD. Pioglitazone decreased microglia and astrocyte activation and reduced the number of iNOS-positive cells in the CNS [47]. In trauma, macrophages, and neutrophils are involved in the early stages of inflammation followed by leukocyte recruitment via VCAM-1, ICAM-1, IL-6, IL-8, and COX-2 [75]. The leukocytes and microglia mount inflammatory responses with elevated expression of cytokines, chemokines, adhesion molecules, iNOS, COX-2, and other inflammatory mediators that exacerbate the tissue damage [61]. Treatment with pioglitazone significantly reduced the induction of inflammatory genes, IL-1*β*, IL-6, monocyte chemoatractant protein-1, and intracellular adhesionmolecule-1. The PPAR*γ* antagonist, 2-chloro-5-nitro-*N*-phenyl-benzamide (GW9662), prevented the neuroprotective effect of pioglitazone [48], suggesting the involvement of PPAR*γ*-dependent mechanisms in the regulation of inflammation and new therapeutic avenue for the treatment of MS.

The expression and activation of PPAR*α* in T lymphocytes decreases IL-2 production and proliferation. PPAR*α*-null mice show an augmented LPS-induced inflammatory response and oral treatment with gemfibrozil reduced CD4+ lymphocyte and macrophage infiltration into the CNS of mice with EAE. Several agonists of PPAR*α*, including gemfibrozil and ciprofibrate, decreased murine lymphocyte proliferation in a concentration-dependent manner, in vitro [60]. The gemfibrozil and ciprofibrate-induced IL-4 production in murine and human lymphocytes, whereas IFN*γ* production was decreased. WY14,643, a synthetic PPAR*α* agonist, reduced the IgG response in mice with EAE and impaired generation of IFN*γ*, TNF*α*, and IL-6 in response to MOG peptide in vitro. PPAR*α* and PPAR*β*/*δ* are expressed in astrocytes, while the latter are present more in oligodendrocytes, thus playing a role in the process of remyelination [66]. In AD, the neuroinflammatory components include resident microglia, astrocyte, the complement system, cytokines, and chemokines. Microglia and astrocytes generate beta-amyloid protein that stimulate proinflammatory cytokines in AD brain. PPAR*α* agonists inhibitA*β*-stimulated expression of TNF*α* and IL-6 in a dose dependent manner [40, 42]. In trauma and spinal cord injury-induced edema, neutrophil infiltration and immunoreactivity to TNF*α* were augmented with a worsened recovery of limb function in PPAR*α* knockout than wild type mice. CNS injury leads to rapid recruitment of microglia, macrophage, and astrocytes that secrete IL-1, TNF*α*, iNOS, PGs, and COX-2 [63]. Fenofibrate promotes neurological recovery by decreasing iNOS, COX2, MMP9 expression, and antioxidant effect in TBI. Although PPAR agonists inhibit neuroinflammation in many CNS diseases, their modes of action are not well characterized.

## 6. PPAR AGONISTS REGULATE IL-12 FAMILY CYTOKINES IN CNS DISEASES

**Figure 2:**
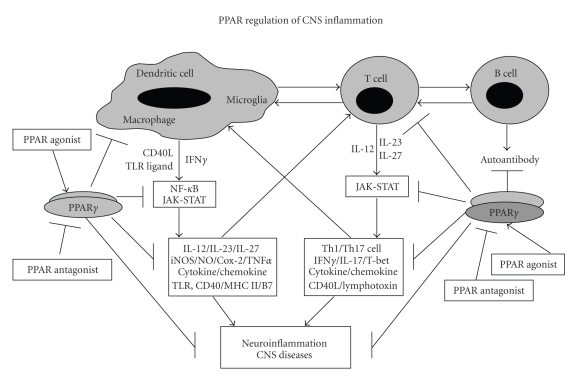
Regulation of neuroinflammation by PPAR agonists in CNS diseases. CD40/TLR induce the activation of NF-*κ*B pathway leading to expression of IL-12 family cytokines from APCs which in turn signal through Jak-Stat pathway in T cells leading to Th1/Th17 differentiation and development of CNS diseases. PPAR agonists modulate signaling and transcription in APC and T cells thereby preventing CNS diseases.

IL-12, IL-23, and IL-27 are three IL-12 family cytokines produced by macrophage, microglia, and dendritic cells in the CNS. IL-12 is a 70 kD heterodimeric cytokine composed of p40 and p35 subunits encoded by two different genes that play a critical role in the differentiation of neural antigen-specific Th1 cells in EAE [78, 79]. We and others have shown earlier that in vivo treatment with neutralizing anti-IL-12p40 antibody prevents EAE [79]. Furthermore, therapeutic intervention of IL-12-signaling was effective in preventing EAE. We have shown that PPAR*γ* agonists inhibit IL-12 production, IL-12 signaling, and differentiation of Th1 cells in EAE [30]. We have also shown that PPAR*γ*-deficient heterozygous mice develop an exacerbated EAE in association with an augmented Th1 response [37], suggesting a physiological role for PPAR*γ* in the regulation of IL-12/IFN*γ* axis in CNS demyelination. IL-23 is a heterodimeric cytokine composed of a common IL-12p40 subunit and an IL-23p19 subunit specific to IL-23 encoded by two different genes [70]. Signaling through its receptor, composed of IL-12R*β*1 and IL-23R, IL-23 induces the activation of Jak-Stat pathways and differentiation of IL-17 producing (Th17) cells from memory Th1 cells, leading to the pathogenesis of EAE [80]. Targeted disruption of IL-23p19 in mice was effective in preventing the pathogenesis of EAE [70, 81] and suggested that the IL-23/IL-17 axis plays a critical role in the pathogenesis of CNS inflammation and demyelination. Although IL-6 and TGF*β* [82] are important mediators of Th17 differentiation in culture, their physiological role in activating Th17 cells in CNS disease is not known (Figure 2).

IL-27 is another heterodimeric cytokine consisting of EBI3 and p28 encoded by two different genes. IL-27 receptor is composed of WSX-1 and gp130 molecules that mediate IL-27-induced activation of the Jak-Stat pathway in naive CD4+ T cells [83]. In vivo treatment with anti-IL-27 antibody ameliorates EAE, suggesting its role in the pathogenesis of Th1 cell-mediated autoimmune diseases. Recent studies have also shown that IL-27 and IFN*γ* are potent inducers of T-bet, a T-box protein transcription factor, in T cells. Targeted disruption of T-bet or siRNA inhibition of T-bet was sufficient to prevent the pathogenesis of EAE, suggesting the critical role of IL-27/IFN*γ*/T-bet axis in the pathogenesis of demyelination [84]. PPAR*γ* agonists regulate IL-27/IFN*γ*/T-bet axis in EAE. Interestingly, recent studies have shown that EBI3 can also heterodimerize with IL-12p35 to form IL-35 in CD4+-Foxp3+ regulatory T cells and functions as a potent anti-inflammatory cytokine [85]. Although PPAR*γ* has been shown to upregulate Treg cells in vitro [86], the role of PPAR in the development of Treg cells or production of IL-35 in EAE/MS or other CNS diseases is not known.

## 7. PPAR AGONISTS REGULATE NF-*κ*B SIGNALING PATHWAYS IN CNS DISEASES

**Figure 3:**
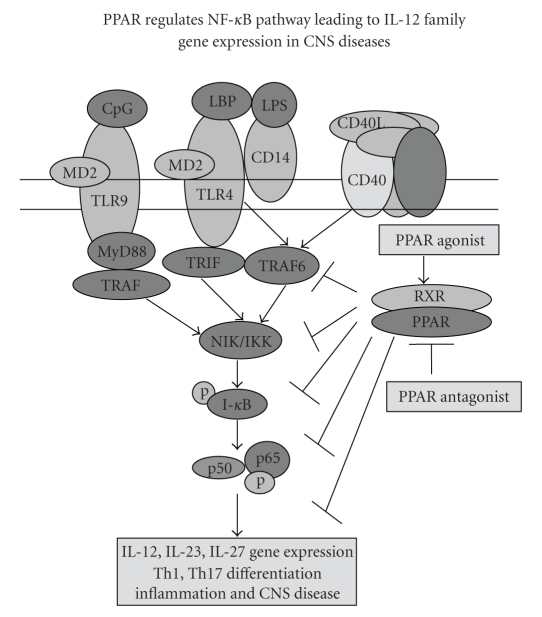
Regulation of NF-*κ*B pathway by PPAR agonists in CNS diseases. The activation of microglia, macrophage and dendritic cells through toll-like receptor, CD40 or cytokine associated NF-*κ*B pathway leads to secretion of inflammatory cytokines leading to pathogenesis of CNS diseases. PPAR agonists inhibit NF-*κ*B pathway resulting in inhibition of CNS diseases.

The IL-12 family cytokines are produced by macrophage, microglia, and dendritic cells in response to autoantigens, TLR ligands, and CD40 ligands [87]. In earlier studies, we and others have shown that autoimmune cells secrete IL-12 in response to antigens and that this response was inhibited by treatment with PPAR*γ* agonists [30]. PPAR*γ* agonists also inhibit LPS and CD40L-induced secretion of IL-12 from macrophage, microglia, and dendritic cells. The induction of IL-12/IL-23 gene expression involves activation of the NF-*κ*B signaling pathway in antigen-presenting cells [88]. NF-*κ*B is a heterodimeric transcription factor composed of p50 and p65 subunits from the Rel family of proteins. It is sequestered in the cytoplasm as an inactive complex when associated with its inhibitor, I*κ*B. Upon stimulation with specific inducers, I*κ*B is phosphorylated and degraded through proteosome-mediated pathways. The activated NF-*κ*B then translocates into the nucleus and binds to specific 10 bp response elements of the IL-12, IL-23, and IL-27 genes [88, 89] Activation of NF-*κ*B is a complex process involving the successive action of proximal NF-*κ*B-inducing kinase (NIK) and the I*κ*B kinases, IKK*α*, IKK*β*, and IKK*γ* [90]. The expression of the IL-12 p40 subunit is controlled by proximal cis-acting elements (NF-*κ*B half site) interacting with NF-*κ*B family members [91]. Inhibitors of IL-12 gene expression, including retinoids, acetyl salicylic acid, and 1,25 dihydroxyvitamin D3, block NF-*κ*B activation and bind within the IL-12p40 promoter [92, 93]. The inhibition of NF-*κ*B pathway leading to the expression of IL-12 family cytokines by PPAR agonists suggests this be a mechanism by which PPAR agonists regulate CNS diseases (Figure 3).

The NF-*κ*B family of proteins (RelA/p65, RelB, c-Rel, p50, p52) are widely expressed in the CNS [94] and activated in a number of CNS inflammatory diseases. Microglia plays a pivotal role in immune surveillance and host defense against infectious agents in the CNS. NF-*κ*B, JNK, and p38 pathways are responsible for F-actin architecture during microglial activation. In AD, NF-*κ*B activation is increased when compared to control brain. The brain samples from PD patients showed an increased nuclear p65 (RelA) in dopaminergic neurons when compared to age matched controls [95]. The spinal cord samples from ALS patients with degenerating motor neurons showed increased NF-*κ*B activation in astrocytes that are controlled by c-jun, and JNK/SAPK kinases [96]. In MS patients, NF-*κ*B and c-jun activities are increased in chronic lesions. PPARs are expressed in microglial cells and PPAR*γ* agonists act as negative regulators for elements that contain Stat binding sites. While the inflammatory cascade is mediated via both NF-*κ*B and JNK pathways, PPAR*γ* agonists increase the levels of I*κ*B-*α* and I*κ*B-*β* and reduce the nuclear translocation of NF-*κ*B [97]. While the induction of NF-*κ*B promotes postischemic inflammation, PPAR agonists prevent postischemic inflammation and neuronal damage by inhibiting NF-*κ*B pathway. Further analyses indicate that L-796,449 inhibits NF-*κ*B signaling through both PPAR*γ*-dependent and independent pathways. In addition, spinal cord injury (SCI) associated neuronal damage was less severe in NF-*κ*B knockout mice. PPAR*γ* induces transrepression of NF-*κ*B-induced inflammatory genes through their association with corepressor complexes [96].

## 8. PPAR AGONISTS REGULATE JAK-STAT SIGNALING PATHWAY IN CNS DISEASES

**Figure 4:**
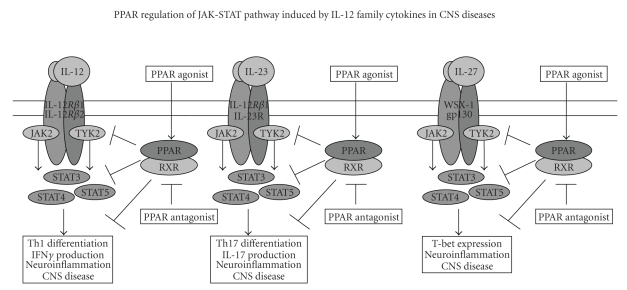
IL-12, IL-23 and IL-27 are heterodimeric cytokines signals through Jak-Stat pathway and induce Th1/Th17 differentiation and T-bet expression in T cells. Treatment with PPAR agonists regulate these responses in T cells resulting in inhibition of CNS inflammation.

The orchestrated interaction of APCs and T cells in the CNS leads to activation of Jak-Stat signaling pathway, secretion of inflammatory cytokines, and pathogenesis of neuroinflammatory diseases. The antigen-induced proliferation of T cells is a two-step process in which signaling through T cell receptor (signal 1) drives T cells from resting G0 to activated G1 phase of the cell cycle, whereas signaling through IL-2 or IL-12 receptor (second signal) is required for T cells to transit from G1 to S/G2/M phase of the cell cycle (proliferation). IL-12 is a potent inducer of G1 to S/G2/M phase transition and differentiation of Th1 cells that are critical in the pathogenesis of EAE and other CNS diseases. IL-12 signals through IL-12 receptor *β*1 and *β*2, members of the gp130 cytokine receptor super-family, expressed primarily on activated NK cells and T cells. Coexpression of IL-12R*β*1 and *β*2 leads to the formation of high affinity IL-12 receptors [87]. Signaling through its receptor, IL-12 induces tyrosine phosphorylation and activation of Jak2, Tyk2, Stat3, and Stat4 in T and NK cells [98, 99]. Activation of the Jak-Stat pathway leads to transcription of IL-12 response genes associated with proliferation, Th1 differentiation, and IFN*γ* production. IL-23 receptor is composed of common IL-12R*β*1 and a specific IL-23 receptor subunit [100]. Signaling through its receptor, IL-23 induces the activation of Jak2, Tyk2, Stat1, Stat3, Stat4, and Stat5 in T cells [98]. Activation of the Jak-Stat pathway leads to transcription of IL-23 response genes, including IL-17, which are associated with proliferation of memory T cells [101], whereas IL-27 and IFN*γ* activate a specific Jak-Stat pathway in T cells, resulting in the induction of T-bet in naive T cells [102]. Modulation of cytokine signaling by targeting protein tyrosine kinases or transcription factors has been considered a novel strategy for the treatment of autoimmune diseases [103, 104]. We have shown earlier that the blockade of IL-12 signaling through Jak-Stat pathway by treatment with a Jak-2 inhibitor, tyrphostin AG490, quercetin, vitamin D, and curcumin inhibits Th1 differentiation and pathogenesis of EAE [105–108]. We have also shown recently that PPAR*γ* agonists inhibit IL-12-induced tyrosine phosphorylation of Jak2, Tyk2, Stat3, and Stat4 in T cells, differentiation of Th1 cells and pathogenesis of EAE [30]. These findings suggest that IL-12 signaling through the Jak-Stat pathway is a molecular target in the regulation of autoimmune diseases. Recent studies have shown that the transcription factors such as Stat4 and T-bet are involved in the pathogenesis of EAE/MS, whereas Stat6 mediates recovery. While the induction of Stat1 and Stat3 promotes postischemic inflammation, and Stat-1 knockout mice develop less severe stroke lesions in the CNS [32], activation of PPARs prevents postischemic inflammation and neuronal damage (Figure 4).

The exact mechanism by which PPAR agonists negatively regulate neuroinflammation, and in particular, the Jak-Stat signaling pathway is not known. Suppressor of cytokine signaling (SOCS) proteins are negative regulators of Jak-Stat pathway. While PPAR*γ* agonists inhibit Jak-Stat pathway in astrocytes and microglial cells, they rapidly induce the expression of SOCS 1 and 3, which in turn inhibit Jak activity in glial cells [109]. In addition, PPAR agonist can modulate Jak-Stat pathway through activation of Src homology 2 domain-containing protein phosphatase 2 (SHP2) in immune cells, thereby inhibiting neuroinflammatory diseases.

## 9. CONCLUSION

The neuroinflammatory diseases such as multiple sclerosis, Alzhimer's disease, stroke, and trauma are common health problems affecting more than five percent of the population worldwide. While the exact mechanisms are not known, the immune cell activation and secretion of inflammatory cytokines, involving NF-*κ*B and Jak-Stat signaling pathways, play critical roles in the pathogenesis of many CNS diseases. Thus, interfering with the signaling network could be an effective approach in the treatment of MS and other neuroinflammatory diseases. PPAR is a family of nuclear receptor transcription factors that regulate CNS diseases by modulating neuroinflammatory signaling network. Since PPAR agonists are already in human use, they are likely to prove useful in the treatment of MS and other neuroinflammatory diseases in the near future.

## Figures and Tables

**Table 1:** Role of PPARs in the regulation of neuroinflammatory diseases.

CNS disease	Inflammatory response	Effect of PPAR agonists
Multiple sclerosis	Activation of macrophage, microglia and dendritic cells; infiltration of Th1/Th17 cells in the CNS; induction of NF-*κ*B and Jak-Stat pathway and release of IL-12, IFN*γ*, IL-17 and other cytokines in the CNS	PPAR*α*, *δ* and *γ* agonists ameliorate EAE by inhibiting inflammation
Alzheimer's disease	Beta-amyloid (A*β*) accumulation leads to CNS inflammation via TNF*α* and NF-*κ*B pathway and secretion of inflammatory cytokines	PPAR*γ* ligands reduce neuronal loss in animal models of AD
Infection	During bacterial, viral, fungal and parasitic infection, activated APC and T cells release TNF*α*, IFN*γ*, iNOS, IL-2, IL-6 and induce inflammation via NF-*κ*B, Stat and AP-1 signaling pathways	PPAR agonists regulate infection associated inflammation
Trauma	CNS injury results in the activation of resident microglia and astrocytes resulting inflammation through secretion of TNF*α*, prostaglandin and COX-2 and mediate inflammation via NF-*κ*B, Stat1 and AP-1 pathways	PPAR*α*, *δ* and *γ* ligands regulate inflammatory response in trauma
Ischemia/stroke	Ischemic stroke associates with recruitment and activation of macrophages and neutrophils via increased expression of VCAM-1, ICAM-1, IL-6, IL-8 and COX-2 through Stat-1	PPAR*γ* ligands reduce the infarct size in animal models

**Table 2:** Role of PPARs in the regulation of inflammatory signaling pathways in CNS diseases.

Tissue Distribution	PPAR Agonists	Effect and Mode of Action in CNS diseases
*PPAR* *α* Expressed in liver, heart, kidney, large intestine, skeletal muscle and astrocytes. PPAR*α* knockout mice develop severe LPS-induced inflammation	Palmitic acid, linoleic acid, stearic acid, palmitoleic acid, oleic acid, 8-HETE, Wy-14643, clofibrate, nafenopin, bezafibrate, fenofibrate	PPAR*α* agonists inhibit A*β* induced expression of TNF*α*, IL-6, IL-4 and infiltration of CD4^+^ T cells in the CNS of AD; reduce ICAM-1 expression and oxidative damage in stroke; protect MPTP-induced loss of neurons in PD; protect mice from EAE by inhibiting IFN*γ*, TNF*α* and IL-6 production in stroke, cerebral ischemia and MS models
*PPAR* *β*/*δ* Expressed ubiquitously in brain, adipose tissue and skin. PPAR*β*/*δ* knockout mice show reduced fat deposition	Prostacyclin, PGI2, GW0742, GW501516, GW7842, L165041	PPAR*β*/*δ* agonists reduce the severity of EAE and stroke by inhibiting NF-*κ*B and Jak-Stat signaling pathways in immune cells from MS and stroke models
*PPAR* *γ* Expressed in heart, muscle, colon, kidney, pancreas, spleen, macrophage, intestine, adipose tissue and liver. PPAR*γ* knockouts are embryonically lethal	Prostaglandin J2, thiazolidinediones, pioglitazone, rosiglitazone, GW78456, WY14,643, GW7647	PPAR*γ* agonists inhibit T-cell proliferation, IFN*γ*, IL-10 and IL-4 production through blocking NF-*κ*B, AP-1 and Jak-Stat pathways in CNS diseases models of AD, PD, Trauma, MS, ALS, stroke and ischemia

## References

[1] Mustafa MM, Ramilo O, Olsen KD (1989). Tumor necrosis factor in mediating experimental Haemophilus influenzae type B meningitis. *The Journal of Clinical Investigation*.

[2] Maffei CML, Mirels LF, Sobel RA, Clemons KV, Stevens DA (2004). Cytokine and inducible nitric oxide synthase mRNA expression during experimental murine cryptococcal meningoencephalitis. *Infection and Immunity*.

[3] Dotis J, Roilides E (2007). Immunopathogenesis of central nervous system fungal infections. *Neurology India*.

[4] Lucchinetti C, Brück W, Noseworthy J (2001). Multiple sclerosis: recent developments in neuropathology, pathogenesis, magnetic resonance imaging studies and treatment. *Current Opinion in Neurology*.

[5] Becher B, Bechmann I, Greter M (2006). Antigen presentation in autoimmunity and CNS inflammation: how T lymphocytes recognize the brain. *Journal of Molecular Medicine*.

[6] Hemmer B, Archelos JJ, Hartung H-P (2002). New concepts in the immunopathogenesis of multiple sclerosis. *Nature Reviews Neuroscience*.

[7] Khan TK, Alkon DL (2006). An internally controlled peripheral biomarker for Alzheimer's disease: Erk1 and Erk2 responses to the inflammatory signal bradykinin. *Proceedings of the National Academy of Sciences of the United States of America*.

[8] Longo FM, Massa SM (2004). Neuroprotective strategies in Alzheimer's disease. *Journal of the American Society of Experimental Neurotherapeutics*.

[9] Jones T, Ugalde V, Franks P, Zhou H, White RH (2005). Venous thromboembolism after spinal cord injury: incidence, time course, and associated risk factors in 16,240 adults and children. *Archives of Physical Medicine and Rehabilitation*.

[10] Jones TB, Hart RP, Popovich PG (2005). Molecular control of physiological and pathological T-cell recruitment after mouse spinal cord injury. *Journal of Neuroscience*.

[11] Wingerchuk DM, Lucchinetti CF, Noseworthy JH (2001). Multiple sclerosis: current pathophysiological concepts. *Laboratory Investigation*.

[12] Blumberg B, Evans RM (1998). Orphan nuclear receptors—new ligands and new possibilities. *Genes & Development*.

[13] Uppenberg J, Svensson C, Jaki M, Bertilsson G, Jendeberg L, Berkenstam A (1998). Crystal structure of the ligand binding domain of the human nuclear receptor PPAR*γ*. *The Journal of Biological Chemistry*.

[14] Shao D, Rangwala SM, Bailey ST, Krakow SL, Reginato MJ, Lazar MA (1998). Interdomain communication regulating ligand binding by PPAR-*γ*. *Nature*.

[15] Kliewer SA, Umesono K, Noonan DJ, Heyman RA, Evans RM (1992). Convergence of 9-*cis* retinoic acid and peroxisome proliferator signalling pathways through heterodimer formation of their receptors. *Nature*.

[16] Nolte RT, Wisely GB, Westin S (1998). Ligand binding and co-activator assembly of the peroxisome proliferator-activated receptor-*γ*. *Nature*.

[17] Janknecht R, Hunter T (1996). A growing coactivator network. *Nature*.

[18] Nuclear Receptors Nomenclature Committee (1999). A unified nomenclature system for the nuclear receptor superfamily. *Cell*.

[19] Murakami K, Tobe K, Ide T (1998). A novel insulin sensitizer acts as a coligand for peroxisome proliferator-activated receptor-*α* (PPAR-*α*) and PPAR-*γ*. Effect of PPAR-*α* activation on abnormal lipid metabolism in liver of Zucker fatty rats. *Diabetes*.

[20] Krey G, Braissant O, L'Horset F (1997). Fatty acids, eicosanoids, and hypolipidemic agents identified as ligands of peroxisome proliferator-activated receptors by coactivator-dependent receptor ligand assay. *Molecular Endocrinology*.

[21] Peters JM, Lee SST, Li W (2000). Growths, adipose, brain, and skin alterations resulting from targeted disruption of the mouse peroxisome proliferator-activated receptor *β*(*δ*). *Molecular and Cellular Biology*.

[22] Saluja I, Granneman JG, Skoff RP (2001). PPAR *δ* agonists stimulate oligodendrocyte differentiation in tissue culture. *Glia*.

[23] Polak PE, Kalinin S, Dello Russo C (2005). Protective effects of a peroxisome proliferator-activated receptor-*β*/*δ* agonist in experimental autoimmune encephalomyelitis. *Journal of Neuroimmunology*.

[24] Pialat J-B, Cho T-H, Beuf O (2007). MRI monitoring of focal cerebral ischemia in peroxisome proliferator-activated receptor (PPAR)-deficient mice. *NMR in Biomedicine*.

[25] Harrington WW, Britt CS, Wilson JG (2007). The effect of PPAR*α*, PPAR*δ*, PPAR*γ*, and PPARpan agonists on body weight, body mass, and serum lipid profiles in diet-induced obese AKR/J Mice. *PPAR Research*.

[26] Barak Y, Nelson MC, Ong ES (1999). PPAR*γ* is required for placental, cardiac, and adipose tissue development. *Molecular Cell*.

[27] Storer PD, Xu J, Chavis J, Drew PD (2005). Peroxisome proliferator-activated receptor-gamma agonists inhibit the activation of microglia and astrocytes: implications for multiple sclerosis. *Journal of Neuroimmunology*.

[28] Storer PD, Xu J, Chavis JA, Drew PD (2005). Cyclopentenone prostaglandins PGA_2_ and 15-deoxy-Δ^12,14^PGJ_2_ suppress activation of marine microglia and astrocytes: implications for multiple sclerosis. *Journal of Neuroscience Research*.

[29] Dey D, Medicherla S, Neogi P (2003). A novel peroxisome proliferator-activated gamma (PPAR*γ*) agonist, CLX-0921, has potent antihyperglycemic activity with low adipogenic potential. *Metabolism*.

[30] Natarajan C, Bright JJ (2002). Peroxisome proliferator-activated receptor-gamma agonist inhibit experimental allergic encephalomyelitis by blocking IL-12 production, IL-12 signaling and Th1 differentiation. *Genes & Immunity*.

[31] Diab A, Deng C, Smith JD (2002). Peroxisome proliferator-activated receptor-*γ* agonist 15-deoxy-Δ^12,14^-prostaglandin J_2_ ameliorates experimental autoimmune encephalomyelitis. *The Journal of Immunology*.

[32] Feinstein DL, Galea E, Gavrilyuk V (2002). Peroxisome proliferator-activated receptor-*γ* agonists prevent experimental autoimmune encephalomyelitis. *Annals of Neurology*.

[33] Storer PD, Xu J, Chavis J, Drew PD (2005). Peroxisome proliferator-activated receptor-gamma agonists inhibit the activation of microglia and astrocytes: implications for multiple sclerosis. *Journal of Neuroimmunology*.

[34] Niino M, Iwabuchi K, Kikuchi S (2001). Amelioration of experimental autoimmune encephalomyelitis in C57BL/6 mice by an agonist of peroxisome proliferator-activated receptor-*γ*. *Journal of Neuroimmunology*.

[35] Schmidt S, Moric E, Schmidt M, Sastre M, Feinstein DL, Heneka MT (2004). Anti-inflammatory and antiproliferative actions of PPAR-*γ* agonists on T lymphocytes derived from MS patients. *Journal of Leukocyte Biology*.

[36] Klotz L, Schmidt M, Giese T (2005). Proinflammatory stimulation and pioglitazone treatment regulate peroxisome proliferator-activated receptor *γ* levels in peripheral blood mononuclear cells from healthy controls and multiple sclerosis patients. *The Journal of Immunology*.

[37] Bright JJ, Natarajan C, Muthian G, Barak Y, Evans RM (2003). Peroxisome proliferator-activated receptor-*γ*-deficient heterozygous mice develop an exacerbated neural antigen-induced Th1 response and experimental allergic encephalomyelitis. *The Journal of Immunology*.

[38] Raikwar HP, Muthian G, Rajasingh J, Johnson CN, Bright JJ (2006). PPAR*γ* antagonists reverse the inhibition of neural antigen-specific Th_1_ response and experimental allergic encephalomyelitis by Ciglitazone and 15-deoxy-Δ^12,14^-Prostaglandin J_2_. *Journal of Neuroimmunology*.

[39] Rosenberg PB (2005). Clinical aspects of inflammation in Alzheimer's disease. *International Review of Psychiatry*.

[40] Hirohata M, Ono K, Naiki H, Yamada M (2005). Non-steroidal anti-inflammatory drugs have anti-amyloidogenic effects for Alzheimer's *β*-amyloid fibrils in vitro. *Neuropharmacology*.

[41] McTigue DM, Tripathi R, Wei P, Lash AT (2007). The PPAR gamma agonist Pioglitazone improves anatomical and locomotor recovery after rodent spinal cord injury. *Experimental Neurology*.

[42] Heneka MT, Sastre M, Dumitrescu-Ozimek L (2005). Acute treatment with the PPAR*γ* agonist pioglitazone and ibuprofen reduces glial inflammation and A*β*1-42 levels in APPV717I transgenic mice. *Brain*.

[43] Watson GS, Cholerton BA, Reger MA (2005). Preserved cognition in patients with early Alzheimer disease and amnestic mild cognitive impairment during treatment with rosiglitazone: a preliminary study. *American Journal of Geriatric Psychiatry*.

[44] Wahner AD, Bronstein JM, Bordelon YM, Ritz B (2007). Nonsteroidal anti-inflammatory drugs may protect against Parkinson disease. *Neurology*.

[45] Casper D, Yaparpalvi U, Rempel N, Werner P (2000). Ibuprofen protects dopaminergic neurons against glutamate toxicity in vitro. *Neuroscience Letters*.

[46] Wahner AD, Bronstein JM, Bordelon YM, Ritz B (2007). Nonsteroidal anti-inflammatory drugs may protect against Parkinson disease. *Neurology*.

[47] Dobrian D, Schriver SD, Khraibi AA, Prewitt RL (2004). Pioglitazone prevents hypertension and reduces oxidative stress in diet-induced
obesity. *Hypertension*.

[48] Schütz B, Reimann J, Dumitrescu-Ozimek L (2005). The oral antidiabetic pioglitazone protects from neurodegeneration and amyotrophic lateral sclerosis-like symptoms in superoxide dismutase-G93A transgenic mice. *Journal of Neuroscience*.

[49] Deplanque D (2004). Cell protection through PPAR nuclear receptor activation. *Thérapie*.

[50] Culman J, Zhao Y, Gohlke P, Herdegen T (2007). PPAR-*γ*: therapeutic target for ischemic stroke. *Trends in Pharmacological Sciences*.

[51] Fujimoto ST, Longhi L, Saatman KE, McIntosh TK (2004). Motor and cognitive function evaluation following experimental traumatic brain injury. *Neuroscience & Biobehavioral Reviews*.

[52] Lee B-C, Doo H-K, Ahn S-Y (2007). Peroxisome proliferator-activated receptor-*γ* Pro12Ala polymorphism is associated with the susceptibility to ischemic stroke in Taeeumin classified by Sasang medicine. *Neurological Research*.

[53] Allahtavakoli M, Shabanzadeh AP, Sadr SS, Parviz M, Djahanguiri B (2006). Rosiglitazone, a peroxisome proliferator-activated receptor-*γ* ligand, reduces infarction volume and neurological deficits in an embolic model of stroke. *Clinical and Experimental Pharmacology and Physiology*.

[54] Ou Z, Zhao X, Labiche LA (2006). Neuronal expression of peroxisome proliferator-activated receptor-gamma (PPAR*γ*) and 15d-prostaglandin J_2_—mediated protection of brain after experimental cerebral ischemia in rat. *Brain Research*.

[55] Zhao Y, Patzer A, Gohlke P, Herdegen T, Culman J (2005). The intracerebral application of the PPAR*γ*-ligand pioglitazone confers neuroprotection against focal ischaemia in the rat brain. *European Journal of Neuroscience*.

[56] Tureyen K, Kapadia R, Bowen KK (2007). Peroxisome proliferator-activated receptor-*γ* agonists induce neuroprotection following transient focal ischemia in normotensive, normoglycemic as well as hypertensive and type-2 diabetic rodents. *Journal of Neurochemistry*.

[57] Pereira MP, Hurtado O, Cárdenas A (2005). The nonthiazolidinedione PPAR*γ* agonist L-796,449 is neuroprotective in experimental stroke. *Journal of Neuropathology & Experimental Neurology*.

[58] Nakamura T, Yamamoto E, Kataoka K (2007). Pioglitazone exerts protective effects against stroke in stroke-prone spontaneously hypertensive rats, independently of blood pressure. *Stroke*.

[59] Lincoff AM, Wolski K, Nicholls SJ, Nissen SE (2007). Pioglitazone and risk of cardiovascular events in patients with type 2 diabetes mellitus: a meta-analysis of randomized trials. *The Journal of the American Medical Association*.

[60] Dasgupta S, Roy A, Jana M, Hartley DM, Pahan K (2007). Gemfibrozil ameliorates relapsing-remitting experimental autoimmune encephalomyelitis independent of peroxisome proliferator-activated receptor-*α*. *Molecular Pharmacology*.

[61] Deplanque D, Gelé P, Pétrault O (2003). Peroxisome proliferator-activated receptor-*α* activation as a mechanism of preventive neuroprotection induced by chronic fenofibrate treatment. *The Journal of Neuroscience*.

[62] Collino M, Aragno M, Mastrocola R (2006). Oxidative stress and inflammatory response evoked by transient cerebral ischemia/reperfusion: effects of the PPAR-*α* agonist WY14643. *Free Radical Biology and Medicine*.

[63] Besson VC, Chen XR, Plotkine M, Marchand-Verrecchia C (2005). Fenofibrate, a peroxisome proliferator-activated receptor *α* agonist, exerts neuroprotective effects in traumatic brain injury. *Neuroscience Letters*.

[64] Gül Ş, Çelik SE, Kalayci M, Taşyürekli M, Çokar N, Bilge T (2005). Dose-dependent neuroprotective effects of melatonin on experimental spinal cord injury in rats. *Surgical Neurology*.

[65] Haghighi SS, Agrawal SK, Surdell D (2000). Effects of methylprednisolone and MK-801 on functional recovery after experimental chronic spinal cord injury. *Spinal Cord*.

[66] Polak PE, Kalinin S, Dello Russo C (2005). Protective effects of a peroxisome proliferator-activated receptor-*β*/*δ* agonist in experimental autoimmune encephalomyelitis. *Journal of Neuroimmunology*.

[67] Iwashita A, Muramatsu Y, Yamazaki T (2007). Neuroprotective efficacy of the peroxisome proliferator-activated receptor *δ*-selective agonists in vitro and in vivo. *Journal of Pharmacology and Experimental Therapeutics*.

[68] Leonard JP, Waldburger KE, Goldman SJ (1995). Prevention of experimental autoimmune encephalomyelitis by antibodies against interleukin 12. *Journal of Experimental Medicine*.

[69] Bright JJ, Musuro BF, Du C, Sriram S (1998). Expression of IL-12 in CNS and lymphoid organs of mice with experimental allergic encephalitis. *Journal of Neuroimmunology*.

[70] Cua DJ, Sherlock J, Chen Y (2003). Interleukin-23 rather than interleukin-12 is the critical cytokine for autoimmune inflammation of the brain. *Nature*.

[71] Szczuciński A, Losy J (2007). Chemokines and chemokine receptors in multiple sclerosis. Potential targets for new therapies. *Acta Neurologica Scandinavica*.

[72] Sindern E (2004). Role of chemokines and their receptors in the pathogenesis of multiple sclerosis. *Frontiers in Bioscience*.

[73] Glabinski AR, Ransohoff RM (2001). Targeting the chemokine system for multiple sclerosis treatment. *Current Opinion in Investigational Drugs*.

[74] Muller DM, Pender MP, Greer JM (2004). Chemokines and chemokine receptors: potential therapeutic targets in multiple sclerosis. *Current Drug Targets: Inflammation & Allergy*.

[75] Ukkonen M, Wu X, Reipert B, Dastidar P, Elovaara I (2007). Cell surface adhesion molecules and cytokine profiles in primary progressive multiple sclerosis. *Multiple Sclerosis*.

[76] Pershadsingh HA, Heneka MT, Saini R, Amin NM, Broeske DJ, Feinstein DL (2004). Effect of pioglitazone treatment in a patient with secondary multiple sclerosis. *Journal of Neuroinflammation*.

[77] Redondo S, Ruiz E, Santos-Gallego CG, Padilla E, Tejerina T (2005). Pioglitazone induces vascular smooth muscle cell apoptosis through a peroxisome proliferator-activated receptor-*γ*, transforming growth factor-*β*1, and a Smad2-dependent mechanism. *Diabetes*.

[78] Shevach EM, Chang JT, Segal BM (1999). The critical role of IL-12 and the IL-12R*β*2 subunit in the generation of pathogenic autoreactive Th1 cells. *Springer Seminars in Immunopathology*.

[79] Segal BM, Dwyer BK, Shevach EM (1998). An interleukin (IL)-10/IL-12 immunoregulatory circuit controls susceptibility to autoimmune disease. *Journal of Experimental Medicine*.

[80] Watford WT, Hissong BD, Bream JH, Kanno Y, Muul L, O'Shea JJ (2004). Signaling by IL-12 and IL-23 and the immunoregulatory roles of STAT4. *Immunological Reviews*.

[81] Touil T, Fitzgerald D, Zhang G-X, Rostami AM, Gran B (2006). Pathophysiology of interleukin-23 in experimental autoimmune encephalomyelitis. *Drug News & Perspectives*.

[82] Veldhoen M, Hocking RJ, Atkins CJ, Locksley RM, Stockinger B (2006). TGF*β* in the context of an inflammatory cytokine milieu supports de novo differentiation of IL-17-producing T cells. *Immunity*.

[83] Kamiya S, Owaki T, Morishima N, Fukai F, Mizuguchi J, Yoshimoto T (2004). An indispensable role for STAT1 in IL-27-induced T-bet expression but not proliferation of naive CD4^+^ T cells. *The Journal of Immunology*.

[84] Gocke AR, Cravens PD, Ben L-H (2007). T-bet regulates the fate of Th1 and Th17 lymphocytes in autoimmunity. *The Journal of Immunology*.

[85] Collison LW, Workman CJ, Kuo TT (2007). The inhibitory cytokine IL-35 contributes to regulatory T-cell function. *Nature*.

[86] Wohlfert EA, Nichols FC, Nevius E, Clark RB (2007). Peroxisome proliferator-activated receptor *γ* (PPAR*γ*) and immunoregulation: enhancement of regulatory T cells through PPAR*γ*-dependent and -independent mechanisms. *The Journal of Immunology*.

[87] Trinchieri G, Pflanz S, Kastelein RA (2003). The IL-12 family of heterodimeric cytokines: new players in the regulation of T cell responses. *Immunity*.

[88] Ghosh S, May MJ, Kopp EB (1998). NF-*κ*B and rel proteins: evolutionarily conserved mediators of immune responses. *Annual Review of Immunology*.

[89] Sha WC (1998). Regulation of immune responses by NF-*κ*B/Rel transcription factors. *Journal of Experimental Medicine*.

[90] Woronicz JD, Gao X, Cao Z, Rothe M, Goeddel DV (1997). I*κ*B kinase-*β*: NF-*κ*B activation and complex formation with I*κ*B kinase-*α* and NIK. *Science*.

[91] Murphy TL, Cleveland MG, Kulesza P, Magram J, Murphy KM (1995). Regulation of interleukin 12 p40 expression through an NF-*κ*B half-site. *Molecular and Cellular Biology*.

[92] Mazzeo D, Panina-Bordignon P, Recalde H, Sinigaglia F, D'Ambrosio D (1998). Decreased IL-12 production and Th1 cell development by acetyl salicylic acid-mediated inhibition of NF-*κ*B. *European Journal of Immunology*.

[93] D'Ambrosio D, Cippitelli M, Cocciolo MG (1998). Inhibition of IL-12 production by 1,25-dihydroxyvitamin D_3_. Involvement of NF-kappaB downregulation in transcriptional repression of the p40 gene. *The Journal of Clinical Investigation*.

[94] Grilli M, Memo M (1999). Nuclear factor-*κ*B/Rel proteins: a point of convergence of signalling pathways relevant in neuronal function and dysfunction. *Biochemical Pharmacology*.

[95] Mattson MP, Camandola S (2001). NF-*κ*B in neuronal plasticity and neurodegenerative disorders. *The Journal of Clinical Investigation*.

[96] Migheli A, Piva R, Atzori C, Troost D, Schiffer D (1997). c-Jun, JNK/SAPK kinases and transcription factor NF-*κ*B are selectively activated in astrocytes, but not motor neurons, in amyotrophic lateral sclerosis. *Journal of Neuropathology & Experimental Neurology*.

[97] Dehmer T, Heneka MT, Sastre M, Dichgans J, Schulz JB (2004). Protection by pioglitazone in the MPTP model of Parkinson's disease correlates with I*κ*B*α* induction and block of NF*κ*B and iNOS activation. *Journal of Neurochemistry*.

[98] Jacobson NG, Szabo SJ, Weber-Nordt RM (1995). Interleukin 12 signaling in T helper type 1 (Th1) cells involves tyrosine phosphorylation of signal transducer and activator of transcription (Stat)3 and Stat4. *Journal of Experimental Medicine*.

[99] Bacon CM, Petricoin EF, Ortaldo JR (1995). Interleukin 12 induces tyrosine phosphorylation and activation of STAT4 in human lymphocytes. *Proceedings of the National Academy of Sciences of the United States of America*.

[100] Parham C, Chirica M, Timans J (2002). A receptor for the heterodimeric cytokine IL-23 is composed of IL-12R*β*1 and a novel cytokine receptor subunit, IL-23R. *The Journal of Immunology*.

[101] Aggarwal S, Ghilardi N, Xie M-H, de Sauvage FJ, Gurney AL (2003). Interleukin-23 promotes a distinct CD4 T cell activation state characterized by the production of interleukin-17. *The Journal of Biological Chemistry*.

[102] Murray PJ (2007). The JAK-STAT signaling pathway: Input and output integration. *The Journal of Immunology*.

[103] O'Shea JJ, Park H, Pesu M, Borie D, Changelian P (2005). New strategies for immunosuppression: interfering with cytokines by targeting the Jak/Stat pathway. *Current Opinion in Rheumatology*.

[104] Seidel HM, Lamb P, Rosen J (2000). Pharmaceutical intervention in the JAK/STAT signaling pathway. *Oncogene*.

[105] Bright JJ, Du D, Sriram S (1999). Tyrphostin B42 inhibits IL-12-induced tyrosine phosphorylation and activation of Janus kinase-2 and prevents experimental allergic encephalomyelitis. *The Journal of Immunology*.

[106] Muthian G, Bright JJ (2004). Quercetin, a flavonoid phytoestrogen, ameliorates experimental allergic encephalomyelitis by blocking IL-12 signaling through JAK-STAT pathway in T lymphocyte. *Journal of Clinical Immunology*.

[107] Muthian G, Raikwar HP, Rajasingh J, Bright JJ (2006). 1,25 dihydroxyvitamin-D3 modulates JAK-STAT pathway in IL-12/IFN*γ* axis leading to Th1 response in experimental allergic encephalomyelitis. *Journal of Neuroscience Research*.

[108] Natarajan C, Bright JJ (2002). Curcumin inhibits experimental allergic encephalomyelitis by blocking IL-12 signaling through Janus kinase-STAT pathway in T lymphocytes. *The Journal of Immunology*.

[109] Lee TL, Yeh J, Van Waes C, Chen Z (2006). Epigenetic modification of *SOCS-1* differentially regulates STAT3 activation in response to interleukin-6 receptor and epidermal growth factor receptor signaling through JAK and/or MEK in head and neck squamous cell carcinomas. *Molecular Cancer Therapeutics*.

